# Classifying Elite From Novice Athletes Using Simulated Wearable Sensor Data

**DOI:** 10.3389/fbioe.2020.00814

**Published:** 2020-08-04

**Authors:** Gwyneth B. Ross, Brittany Dowling, Nikolaus F. Troje, Steven L. Fischer, Ryan B. Graham

**Affiliations:** ^1^School of Human Kinetics, Faculty of Health Sciences, University of Ottawa, Ottawa, ON, Canada; ^2^Motus Global, Rockville Centre, New York, NY, United States; ^3^Centre for Vision Research, York University, Toronto, ON, Canada; ^4^Department of Kinesiology, University of Waterloo, Waterloo, ON, Canada

**Keywords:** inertial measurement units, machine learning, artificial intelligence, principal component analysis, pattern recognition, athletics, movement screening

## Abstract

Movement screens are frequently used to identify differences in movement patterns such as pathological abnormalities or skill related differences in sport; however, abnormalities are often visually detected by a human assessor resulting in poor reliability. Therefore, our previous research has focused on the development of an objective movement assessment tool to classify elite and novice athletes’ kinematic data using machine learning algorithms. Classifying elite and novice athletes can be beneficial to objectively detect differences in movement patterns between the athletes, which can then be used to provide higher quality feedback to athletes and their coaches. Currently, the method requires optical motion capture, which is expensive and time-consuming to use, creating a barrier for adoption within industry. Therefore, the purpose of this study was to assess whether machine learning could classify athletes as elite or novice using data that can be collected easily and inexpensively in the field using inertial measurement units (IMUs). A secondary purpose of this study was to refine the architecture of the tool to optimize classification rates. Motion capture data from 542 athletes performing seven dynamic screening movements were analyzed. A principal component analysis (PCA)-based pattern recognition technique and machine learning algorithms with the Euclidean norm of the segment linear accelerations and angular velocities as inputs were used to classify athletes based on skill level. Depending on the movement, using metrics achievable with IMUs and a linear discriminant analysis (LDA), 75.1–84.7% of athletes were accurately classified as elite or novice. We have provided evidence that suggests our objective, data-driven method can detect meaningful differences during a movement screening battery when using data that can be collected using IMUs, thus providing a large methodological advance as these can be collected in the field using sensors. This method offers an objective, inexpensive tool that can be easily implemented in the field to potentially enhance screening, assessment, and rehabilitation in sport and clinical settings.

## Introduction

Movement screens are widely used across many disciplines including in ergonomic, clinical, and athletic settings to identify aberrant movement patterns in hopes of decreasing risk of injury and/or improving performance (Donà et al., 2009; Kritz et al., 2009; Padua et al., 2009; Cook et al., 2014; McCall et al., 2014; McCunn et al., 2016). Most commonly, during a movement screen, an individual’s movement is evaluated based on visual appraisal (McCunn et al., 2016); however, there is agreement within the literature that inter-rater and inter-session (participants tested during two separate sessions) reliability of these subjective movement screens are poor (Onate et al., 2012; Smith et al., 2013; Gulgin and Hoogenboom, 2014). Therefore, our previous research focused on the development and application of an objective framework as a data-driven alternative to objectively classify movement strategies and quality during a movement screen (Ross et al., 2018), known as the Objective Movement Assessment Tool (OMAT).

The previously published technique with optical motion capture, herein referred to as OMAT-OPT, uses principal component analysis (PCA) (Troje, 2002; Federolf et al., 2014; Young and Reinkensmeyer, 2014) in conjunction with linear discriminant analysis (LDA) to objectively differentiate and score whole-body movement patterns between desired binary classifiers (Ross et al., 2018). For OMAT-OPT, the data input into the PCA are time-series trajectories of joint centers and select anatomical markers, representing the whole-body, captured using an optical motion capture system. During a non-sport-specific movement screening battery consisting of seven unique dynamic movements that challenge stability and mobility across all major joints, between 70.7% and 82.9% of athletes were appropriately classified as either elite or novice depending on the movement (Ross et al., 2018). Although OMAT-OPT provides an objective, data-driven method that can detect meaningful movement pattern differences during a movement screening battery for binary classification, it requires optical motion capture technology, which is expensive and time-consuming to set up, capture and post-process data, reducing the accessibility and feasibility of the current technique in clinical, ergonomic, and sport settings (Hadjidj et al., 2013).

The use of wearable systems are increasing in popularity in clinical, sport, and ergonomic settings (Patel et al., 2012; Hadjidj et al., 2013), offering an inexpensive alternative to optical motion capture systems. The wearable systems are easily transportable, require minimal post-processing, are able to collect data in larger capture volumes compared to optical systems, and are immune to problems associated with optical systems such as occlusion and line-of-sight problems (Zhou and Hu, 2008). A common type of sensor used is the inertial measurement unit (IMU). IMUs contain an accelerometer, gyroscope, and magnetometer, allowing measurement of linear accelerations and angular velocities in three axes and the triaxial magnetic fields of the earth. IMUs are susceptible to drift, especially when close to metal, although more robust algorithms are continuously being developed to mitigate these effects (Madgwick et al., 2011; Wittmann et al., 2019), making them more suitable for use in the field.

IMU data have been used to objectively classify movement based on different classifiers during non-sport specific tasks (Sgro et al., 2017; Johnston et al., 2016, 2019; Zago et al., 2019). Machine learning with IMU data as the input has been able to objectively identify children of different motor development levels during a standing long jump (Sgro et al., 2017), rugby players at a higher risk of a sport-related concussion based on a Y-balance test (Johnston et al., 2019), Australian football players at different levels of fatigue during a Y-balance test (Johnston et al., 2016), and to predict change of direction, speed, and mechanical work during cutting maneuvers (Zago et al., 2019), to name a few. Although these studies only looked at a single IMU placed on the low-back of the participant, these findings suggest that IMUs can be used as an inexpensive alternative to optical motion capture to characterize and classify motion.

Although research using machine learning to classify elite and novice athletes is limited, discriminant analysis has been previously used to classify novice, good, and elite rowers during ergometer testing (Smith and Spinks, 1995). The ability to objectively differentiate movement patterns between novice and elite athletes is useful to highlight emergent differences in movement performance. Guided by those differences, coaches can improve quality of feedback to their athletes (Smith and Spinks, 1995). We chose skill level as the dichotomous factor to initially assess due to its likelihood to influence movement quality and performance, with the intention of in the future expanding to sex, sport played, and injury history or risk.

Feature selection approaches and machine learning algorithms may also influence the accuracy of classification between elite and novice athletes using IMU data and are therefore important secondary considerations. Previously, the OMAT-OPT used the first 35 principal component (PC) scores as the input data for the LDA; however, alternative feature selection approaches could provide an objective method to best decide which PC scores to use as input data to maximize classification. Ensemble feature selection, which is based on the same ideology of ensemble supervised classifiers, is a useful approach to evaluate. Ensemble feature selection includes the use of multiple feature selection algorithms to select features and has been found to have greater stability (i.e., less likelihood of features changing if data are added or removed) and better generalizability than using a single feature selection technique (Saeys et al., 2008). In addition, the OMAT currently uses LDA, which was selected due to superior performance during testing. However, it is unknown whether LDA would still garner the highest classification rates when using PC scores selected by an ensemble feature selection approach, rather than the first 35 PC scores and/or when using IMU data. Alternative machine learning algorithms including binary logistic regression (BLR), decision trees (DT), K-nearest neighbors (kNN), naïve Bayes (NB), support vector machine with a linear kernel (SVM), and support vector machine with a radial basis function kernel (RBF) may strengthen classification accuracy relative to our existing LDA approach. As a result, while investigating the utility of IMUs to classify movements between novice and elite athletes, it remains important to concurrently evaluate the underlying machine learning model architecture required to generate the best possible classification.

Therefore, the purpose of this study was to assess the ability of the previously developed framework to differentiate whole-body movement patterns between novice and elite athletes performing a non-sport-specfic movement screening battery when using data extractable from an IMU (i.e., simulated IMU data; OMAT-sIMU), which can be collected easily and inexpensively in the field. Although data in the current study are simulated IMU data based on optical motion capture, this study provides proof-of-concept that IMU-based data can provide enough information to successfully classify athletes’ movement patterns based on skill level. A secondary purpose of this study was to refine the architecture of the OMAT to optimize classification rates by incorporating feature selection and multiple machine learning algorithms (i.e., BLR, DT, KNN, LDA, NB, SVM, and RBF) for both the OMAT-OPT and OMAT-sIMU.

## Materials and Methods

### Participants

Kinematic data were collected on 542 athletes by Motus Global (Rockville Centre, NY, United States). The sample included athletes competing in 11 different sports (i.e., baseball, basketball, soccer, golf, tennis, track and field, squash, cricket, lacrosse, football, or volleyball) and ranging in skill level from recreational to professional (e.g., NBA, MLB, NFL, PGA, FIFA). The athletes were assigned to either the novice or elite group based on previous research that found that those athletes accruing over 10,000 h of deliberate practice are experts in their sport (Helsen et al., 1998; Baker et al., 2003). Therefore, athletes competing at the inter-collegiate, semi-professional, and professional level were considered elite athletes and those competing at less competitive levels (e.g., high-school, youth, recreational, etc.) were considered novices. Before data collection, each athlete read and signed an informed consent form permitting Motus Global to use the data for future research. The Health Sciences Research Ethics Board at the University of Ottawa approved the secondary use of the data for research purposes (file no: H-08-18-1085).

### Protocol

Upon arrival to the Motus Global laboratory, each athlete read and signed an informed consent form, provided information on injury history for the previous 10 years, and had their height (with shoes on) and weight recorded. The athlete was then outfitted with 45 passive, reflective markers (B&L Engineering, Santa Ana, CA) to capture whole-body motion (Mcpherson et al., 2016; Ross et al., 2018). After being outfitted with the markers, the athlete completed a static and dynamic calibration trial (Ross et al., 2018). The static calibration trial was used to develop a whole-body biomechanical model for each athlete.

**FIGURE 1:**
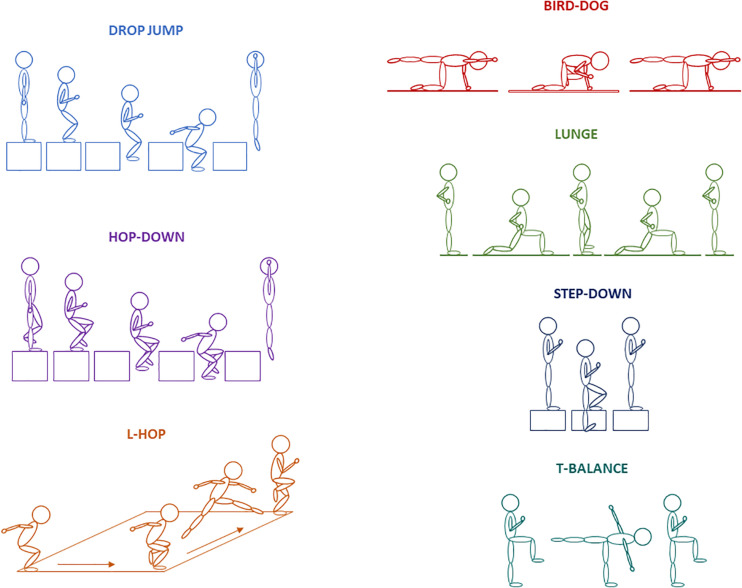
Schematic drawings of the seven unique movements performed by the athletes: drop-jump, hop-down, L-hop, bird-dog, lunge, step-down, and T-balance.

After the calibration trials, each athlete completed a movement battery consisting of 21 unique movements testing athletes’ range of motion at each joint, stability, power and balance. However, only seven movements were used in the analysis due to their dynamic nature and ability to challenge the athletes’ coordination, stability, and mobility across all major joints. The seven tasks included: drop jump, bird-dog, hop-down, lunge, step-down, L-hop, and T-balance (Figure 1). Each movement was performed bilaterally on the left and right side except for the drop jump which was performed symmetrically, resulting in a total of 13 movement trials (Ross et al., 2018). The athlete performed each task until they believed they did it to the best of their ability with only the trial that was deemed the best being retained for each athlete. Full-body motion data were captured at 120 Hz using an 8-camera Raptor-E (Motion Analysis Corporation, Santa Rosa, CA) motion capture system.

### Data Analysis

#### Pre-processing

Motion capture data were collected, labeled, and gap-filled using Cortex (Motion Analysis Corporation, Santa Rosa, CA). Data from anatomical landmarks and the tracking markers during the calibration trial were used to develop a whole-body 3D kinematic model in Visual3D v6 (C-Motion, Inc., Germantown, MD). The model was then applied to all motion trials outputting joint centers bilaterally for the wrist, elbow, shoulder, foot, ankle, knee, and hip; centers of gravity for the trunk, head, and pelvis; marker positional data for the left and right heel, T_2_, T_8_, sternum, and the back, front and sides of the head for the OMAT-OPT model and segment angular velocities and center of gravity (CoG) linear velocities of the head, trunk, pelvis, upper arms, forearms, thighs, shanks, and feet for the OMAT-sIMU model. Data were then exported and analyzed using Python 3.0. All trials were trimmed to specific start and end-point criteria (Ross et al., 2018), and filtered using a dual-pass, low-pass Butterworth filter with a cutoff of 15 Hz. Since elite athletes were significantly taller than novices (*F* = 138.25, *p* < 0.001), all data for each movement were normalized by each athlete’s individual height by dividing each raw data point by their own height. Normalization ensured that differences in PC scores between groups were not strictly due to variation in size.

##### OMAT-OPT data

The 3D positional data of the joint centers and markers retained in the OPT model for each participant were rotated so that the local coordinate system of the trunk was aligned with the global coordinate system. The data were then translated so that the midpoint between the left and right hip of the first frame of data was aligned with the global origin (i.e., midpoint of left and right hip equaled 0,0,0 for x, y, and z coordinates, respectively). The rotated 3D data were then time normalized to 500 frames using Piecewise Cubic Hermite Interpolating Polynomial (PCHIP) interpolation to control for differences in absolute movement time for each participant. An [*n* × 39,000] matrix for each movement was then constructed, where n was the number of subjects and 39,000 was the time-normalized x, y, and z data for each joint center, center of gravity, and retained markers mentioned above (26 positions × 3 axes × 500 time points). Due to marker occlusion and some athletes not performing all tasks, n was dependent on the movement task (Table 1).

**TABLE 1:** OMAT-OPT: The number of athletes broken down by sex and skill level and the overall classification accuracy, hit rate, miss rate, false alarm (FA) rate, correct rejection (CR) rate, D’ and C when the optimal number of PCs were retained for each movement task.

		**Male**		**Female**									
**Movement**	**n**	**Elite**	**Novice**	**Elite**	**Novice**	**# of PCs**	**Accuracy (%)**	**Hit**	**Miss**	**FA**	**CR**	**D’**	**C**
Bird-Dog Left	380	242	83	12	43	10	82.63	0.91	0.09	0.34	0.66	1.75	−0.46
Bird-Dog Right	387	244	88	11	44	18	80.88	0.90	0.10	0.36	0.64	1.62	−0.46
Drop Jump	275	168	64	7	36	12	80.36	0.88	0.12	0.33	0.67	1.61	−0.37
Hop-Down Left	396	242	99	10	45	14	77.27	0.87	0.13	0.40	0.60	1.39	−0.45
Hop-Down Right	396	242	97	11	46	9	74.24	0.84	0.16	0.43	0.57	1.17	−0.40
L-Hop Left	266	159	67	6	34	15	83.83	0.89	0.11	0.25	0.75	1.91	−0.27
L-Hop Right	267	160	67	6	34	14	79.03	0.87	0.13	0.34	0.66	1.54	−0.35
Lunge Left	399	246	97	12	44	11	78.20	0.87	0.13	0.38	0.62	1.43	−0.40
Lunge Right	401	248	97	12	44	17	78.30	0.88	0.12	0.39	0.61	1.44	−0.44
Step-Down Left	399	246	98	12	43	17	75.94	0.84	0.16	0.40	0.60	1.28	−0.38
Step-Down Right	399	247	96	11	45	16	74.19	0.83	0.17	0.42	0.58	1.16	−0.37
T-Balance Left	392	244	92	11	45	13	77.30	0.89	0.11	0.45	0.55	1.37	−0.56
T-Balance Right	395	244	94	12	45	18	73.16	0.83	0.17	0.45	0.55	1.08	−0.41
**Average**	**365.54**	**225.54**	**87.62**	**10.23**	**42.15**	**14.15**	**78.10**	**0.87**	**0.13**	**0.38**	**0.62**	**1.44**	−**0.41**
**STD**	**55.17**	**36.14**	**13.11**	**2.31**	**4.38**	**3.02**	**3.26**	**0.03**	**0.03**	**0.06**	**0.06**	**0.25**	**0.07**

*Bold values represent the average and standard deviation across all movement tasks.*

##### OMAT-sIMU data

In order to simulate IMU accelerometer data, we extracted the segment CoG linear velocities of each segment retained in the model and then differentiated the data once to calculate segment linear accelerations, and to simulate the IMU gyroscope data, we extracted the segment angular velocities of the same segments. Once all data were extracted and calculated, the data were time normalized to 500 frames as per the OMAT-OPT. In anticipation of the future implementation of the method in clinic or industry, the Euclidean norm (i.e., square root of the sum of squares) of the x, y, and z axes of the linear segment accelerations and segment angular velocities were taken to minimize the effect of sensor brand or orientation (Clouthier et al., 2020) and to reduce the dimensionality of the data (Bergmann et al., 2014).

A matrix for each movement was then constructed with the Euclidean norm of the linear segment accelerations and segment angular velocities for each segment and each participant. Segment linear accelerations and angular velocities were chosen to mimic outputs collected via IMUs. Each matrix was n (number of participants; Table 2) × 13000 (Euclidean norm × 2 data features × 13 body segments × 500 time points). Because the units were different between the data features (i.e., linear accelerations in m/s^2^, angular velocities in rad/s), the scale of the data between the two data features varied widely, which would lead to classification being driven primarily by the data feature with the larger scale. Therefore, the data were feature scaled to be between 0 and 1 for each movement using scikit-learn Robust Scaler (Pedregosa et al., 2011), which removes the median from each feature and scales the data according to the 1st and 3rd quartile of the data, mitigating the effect of outliers during scaling.

**TABLE 2:** OMAT-sIMU: The number of athletes broken down by sex and skill level and overall classification accuracy, hit rate, miss rate, false alarm rate, correct rejection rate, D’ and C when the optimal number of PCs were retained for each movement task.

		**Male**		**Female**									
**Movement**	**n**	**Elite**	**Novice**	**Elite**	**Novice**	**# of PCs**	**Accuracy (%)**	**Hit**	**Miss**	**FA**	**CR**	**D’**	**C**
Bird-Dog Left	380	242	83	12	43	6	81.22	0.92	0.08	0.45	0.55	1.56	−0.66
Bird-Dog Right	387	244	88	11	44	7	81.98	0.90	0.10	0.49	0.51	1.34	−0.64
Drop Jump	275	168	64	7	36	14	84.67	0.89	0.11	0.27	0.73	1.81	−0.29
Hop-Down Left	396	242	99	10	45	10	81.75	0.88	0.13	0.36	0.64	1.51	−0.40
Hop-Down Right	396	242	97	11	46	13	79.70	0.89	0.11	0.40	0.60	1.49	−0.50
L-Hop Left	266	159	67	6	34	12	83.15	0.87	0.13	0.29	0.71	1.70	−0.29
L-Hop Right	267	160	67	6	34	15	82.71	0.90	0.10	0.33	0.67	1.71	−0.41
Lunge Left	399	246	97	12	44	18	80.70	0.93	0.07	0.50	0.50	1.47	−0.74
Lunge Right	401	248	97	12	44	18	81.25	0.91	0.09	0.38	0.62	1.62	−0.51
Step-Down Left	399	246	98	12	43	12	75.19	0.87	0.13	0.52	0.48	1.07	−0.60
Step-Down Right	399	247	96	11	45	6	76.37	0.88	0.12	0.50	0.50	1.17	−0.60
T-Balance Left	392	244	92	11	45	14	76.47	0.90	0.10	0.55	0.45	1.15	−0.71
T-Balance Right	395	244	94	12	45	10	75.13	0.87	0.13	0.50	0.50	1.14	−0.58
**Average**	**365.54**	**225.54**	**87.62**	**10.23**	**42.15**	**11.92**	**80.02**	**0.89**	**0.11**	**0.43**	**0.57**	**1.44**	**−0.53**
**STD**	**55.17**	**36.14**	**13.11**	**2.31**	**4.38**	**4.03**	**3.19**	**0.02**	**0.02**	**0.10**	**0.10**	**0.25**	**0.15**

*Bold values represent the average and standard deviation across all movement tasks.*

#### Feature Selection

For both OMAT-sIMU and OMAT-OPT, PCA was applied to each matrix, resulting in a unique model per task per data type (i.e., OMAT-sIMU, OMAT-OPT). Using the PC scores as features, ensemble feature selection, consisting of six common feature selection techniques (Pearson correlation, chi-squared, recursive feature elimination, lasso, random forest, and LightGBM), was used to rank the PCs based on contribution to the model for each movement task and data type. Ensemble feature selection has been found to improve the robustness of feature ranking and feature subset selection as well as increase the generalizability of the features selected (Saeys et al., 2008). The scikit-learn library was used for the chi-squared, recursive feature elimination, lasso, and random forest (Pedregosa et al., 2011). The top 25 features per data type were retained for each technique. The features were then sorted based on the number of techniques where they ranked in the top 25 features. PC scores that ranked in the top 25 for at least 50% of the techniques (i.e., 3) were retained for the classifier (Table 1; OMAT-OPT and 2; OMAT-sIMU). To minimize overfitting of the models, the maximum number of features retained was the square root of the number of samples for each movement task (Hua et al., 2005) (e.g., lunge right had 401 samples, therefore a maximum of 20 PC scores could be retained for that task).

#### Classification

To refine the architecture of the OMAT, seven different kinds of classifiers were used: BLR, DT, kNN, LDA, NB, SVM, and RBF to classify athletes based on skill level (elite vs. novice). All classifiers were employed using the scikit-learn library (Pedregosa et al., 2011). For all classifiers, PC scores retained from feature selection were used as predictors and leave-one-out cross-validation was used for validation. Each model was rerun to use between 1 and the total number of PCs retained to determine the optimal number of PCs to retain for each classifier for each movement task. The model with the highest classification rate was deemed the optimal model. Due to a lack of a testing dataset, leave-one-out validation was used where one of the athletes’ data were taken out (test athlete) and the PCA, feature selection, and classifier models were computed on the remaining athletes (training athletes). After computing the new PCA, feature selection, and classifier models, the test athlete was projected into the PCA, feature selection, and classifier model spaces computed on the training athletes. The procedure was repeated until all athletes had been left out and projected back into the PCA, feature selection, and classifier models (Troje, 2002; Ross et al., 2018).

#### Signal Detection Theory

For the best classifier for each data type, to test the separation between the signal and the noise and to determine the strategy used by the frameworks, a signal detection theory (SDT) model was used for each optimal model retained. In SDT, there are four types of classification: (1) Hit, (2) Miss, (3) False alarm (FA), and (4) Correct rejection (CR) (Abdi, 2007). For this study, a hit was when an elite athlete was correctly classified as an elite (equivalent to sensitivity), a miss was when an elite athlete was misclassified as a novice, a FA was when a novice athlete was misclassified as an elite, and a CR was when a novice athlete was correctly classified as a novice (equivalent to specificity). Parameter D’ is calculated subtracting the probability (z-score) of a false alarm from the probability (z-score) of a hit and tells the distance between the two peaks (e.g., elite and novice) in standard deviations; the higher the score the more separable the two groups are with a score of 0 representing chance (Abdi, 2007). Parameter C is calculated by taking the average probability (z-score) of a hit and false alarm and represents the strategy used by the framework. A positive value represents the framework being conservative (e.g., more likely to classify an athlete as novice), where as a negative value represents the framework being liberal (e.g., more likely to classify an athlete as elite) (Abdi, 2007). The closer the value is to 0, the closer the framework is to being the ideal observer (e.g., not more likely to classify as either elite or novice) (Abdi, 2007).

## Results

### OMAT-OPT

**FIGURE 2:**
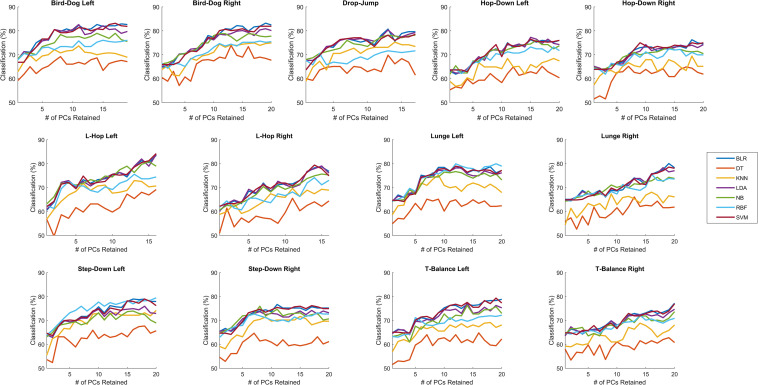
OPT: The percent of correctly classified athletes as either elite or novice for when 1 to the total number of PCs retained were retained for binary logistic regression (BLR), decision tree (DT), linear discriminant analysis (LDA), k-nearest neighbors (kNN), naïve Bayes (NB), support vector machine with a linear kernel (SVM), and support vector machine with a radial basis function kernel (RBF) with leave-one-out validation for OPT. See number of PCs retained in Table 1.

For all tasks the linear classifiers (i.e., BLR, LDA, and SVM) outperformed DT, kNN, and RBF, except RBF performed as well as the linear classifiers for the lunge left and step-down left (Figure 2). For the drop-jump, hop-down left, L-hop left, and lunge left, NB performed as well as the linear classifiers, however, for all other tasks, they performed in between the linear classifiers and DT, kNN, and RBF. Since there were minimal differences (<0.5%) on the average classification rates for all tasks between BLR, LDA, and SVM, and to be able to compare the current results to previous results (Ross et al., 2018), LDA was selected for further analysis. When using LDA, the optimal number of PCs retained ranged from 9 (hop-down right) to 18 (bird-dog left, T-balance right) with an average of 14.15 ± 3.02 PCs retained (Table 1). The OMAT-OPT accurately classified between 73.1% (T-balance right) to 83.8% (L-hop left) of athletes as either elite or novice (Table 1). The average classification rate across all tasks was 78.1% ± 3.26%. For SDT, on average, OMAT-OPT had a hit, miss, FA, and CR rate of 0.87 ± 0.03, 0.13 ± 0.03, 0.38 ± 0.06, and 0.62 ± 0.06, respectively (Table 1). The average D’ was 1.44 ± 0.25 and the average C was −0.41 ± 0.07 (Table 1).

### OMAT-sIMU

**FIGURE 3:**
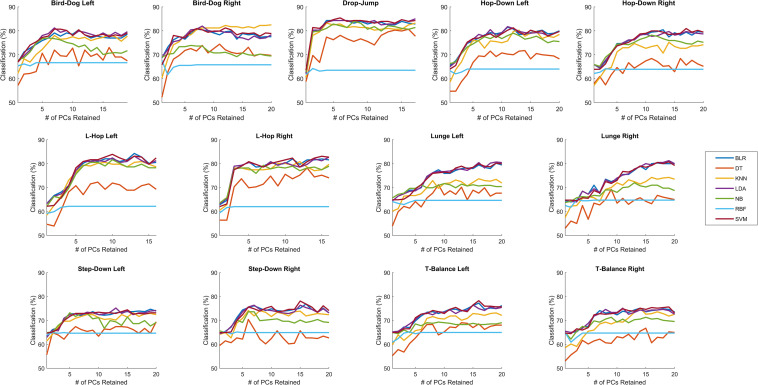
sIMU: The percent of correctly classified athletes as either elite or novice for when 1 to the total number of PCs retained were retained for binary logistic regression (BLR), decision tree (DT), linear discriminant analysis (LDA), k-nearest neighbors (kNN), naïve Bayes (NB), support vector machine with a linear kernel (SVM), and support vector machine with a radial basis function kernel (RBF) with leave-one-out validation for sIMU. See number of PCs retained in Table 2.

Similar to OMAT-OPT, for all tasks the linear classifiers (i.e., BLR, LDA, and SVM) outperformed all the other classifiers (i.e., DT, kNN, NB, and RBF), except KNN performed as well as the linear classifiers in the bird-dog left and right, hop-down left, and step-down left (Figure 3). Since there were again minimal differences between the average classification rates for all movement tasks between BLR, LDA, and SVM, LDA was selected for further analysis. When using segment linear accelerations and angular velocities, data available from an IMU system, the optimal number of PCs retained ranged from 6 (bird-dog left, step-down right) to 18 (lunge left and right) with an average of 11.92 ± 4.03 PCs retained (Table 2). The OMAT-sIMU accurately classified between 75.1% (T-balance right) to 84.7% (drop-jump) of athletes as either elite or novice (Table 2). The average classification rate across all tasks was 80.0% ± 3.19%. For SDT, on average, OMAT-sIMU had a hit, miss, FA, and CR rate of 0.89 ± 0.02, 0.11 ± 0.02, 0.43 ± 0.1, and 0.57 ± 0.1, respectively (Table 2). The average D’ was 1.44 ± 0.25 and the average C was −0.53 ± 0.15 (Table 2).

**FIGURE 4:**
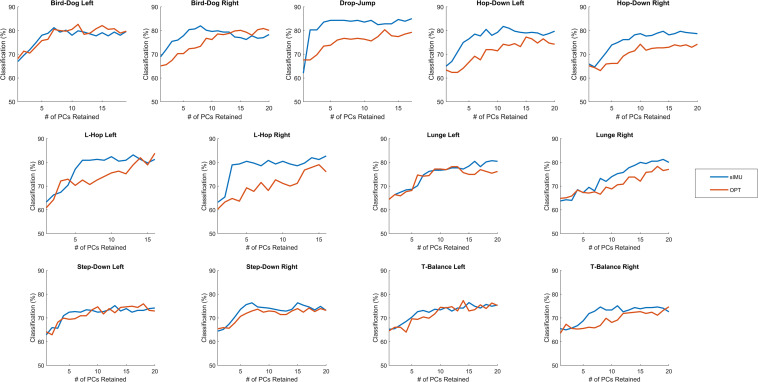
The percent of correctly classified athletes as either elite or novice for when 1 to the total number of PCs retained were retained for the linear discriminant analysis with leave-one-out validation when using OPT and sIMU data.

When comparing the OMAT-OPT and OMAT-sIMU classification rates on average, OMAT-sIMU had higher classification rates than OMAT-OPT by 1.92%. OMAT-sIMU outperformed OMAT-OPT in the bird-dog right (1.1%), drop-jump (4.31%), hop-down left (4.48%) and right (5.46%), L-hop right (3.68%), lunge left (2.50%) and right (2.95%), step-down right (2.18%), and T-balance right (1.97%), whereas OMAT-OPT had a higher classification rate than OMAT-sIMU for bird-dog left (1.41%) (Figure 4). The two models performed relatively the same (< 1% difference) for the L-hop left, step-down left, and T-balance left.

## Discussion

The primary purpose of this study was to assess the ability of the OMAT to differentiate whole-body movement patterns between novice and elite athletes performing a non-sport-specific movement screening battery using data able to be collected via an IMU. The secondary purpose of this study was to refine the architecture of the OMAT by incorporating feature selection and testing multiple classifiers. For both the OMAT-OPT and OMAT-sIMU, BLR, LDA, and SVM, on average, outperformed all other classifiers tested. These findings suggest that the data can be separated using a linear plane; and therefore, the use of more complicated, computationally expensive non-linear classifiers is not only not required, but can be detrimental. There were minimal differences between BLR, LDA, and SVM, so therefore in order to easily compare the current results with previous work, LDA was chosen as the classifier to report the results.

OMAT-OPT with feature selection outperformed the previously published results on 7 of the 13 tasks (i.e., bird-dog left and right, hop-down left, L-hop left, lunge left and right, and T-balance left) and OMAT-sIMU was able to outperform the previously published results of the OMAT-OPT in all tasks except the step-down left (Ross et al., 2018). This in part is due to the introduction of feature selection into the methodology, reinforcing the value of this approach for future work aiming to objectively classify movement patterns. Compared to the previous study, where PCs 1–35 were retained (Ross et al., 2018), using feature selection, we are now able to have greater classification rates using fewer PCs, which requires fewer computational resources and decreases the risk of overfitting for 7 of the 13 tasks. OMAT-sIMU outperformed or performed equally to OMAT-OPT in all movements except the bird-dog left. These findings suggest that the OMAT-sIMU approach better captures movement pattern differences between novice and elite athletes compared to OMAT-OPT data. This is thought to be due to the different types of data analyzed for OMAT-OPT and OMAT-sIMU. OMAT-OPT uses joint center trajectories, which due to the constrained nature of the tasks, may be capturing more gross motor patterns that are unrelated to skill. In contrast, the OMAT-sIMU uses linear acceleration and angular velocity that are more likely to capture the smoothness of the movement, which may be a better indicator of skill level than gross motor patterns. However, for both the OMAT-OPT and OMAT-sIMU, when looking at trends in individual athlete data across tasks, if there were differences in how the athlete was classified between tasks, athletes tended to be classified the same on all tasks that were targeting the same skill set (e.g., trunk stability, jumping, balance) and if there were discrepancies on how the left/right tasks were classified, the dominant side was usually classified as elite. This suggests that relevant differences between elite and novice-like movement patterns can be detected using both data types. A combined approach of using both sIMU and OPT data may provide even better classification rates than using sIMU or OPT alone due to the two types of data potentially capturing different movement features.

Previously, in order to assess how well the framework was classifying elite and novice athletes on a group basis, the percent of correctly classified elites and novices were calculated (Ross et al., 2018). SDT was chosen for this current study because it provides classification rates for each group (e.g., hit and correct rejection) as well as the additional information of response bias. For all tasks, both OMAT-OPT and OMAT-sIMU had higher rates of correctly classifying elite athletes (depicted by the increased hit and decreased miss rates) compared to novice athletes (depicted by the decreased CR and increased FA rates). For all tasks, D’ was greater than 1.08 and 1.07 for OMAT-OPT and OMAT-sIMU, respectively, suggesting that elite and novice athletes are separable when using both OMAT-OPT and OMAT-sIMU. However, on average, the data are more robustly classified when using OMAT-sIMU data compared to OMAT-OPT data. Lastly, for all tasks, for both OMAT-OPT and OMAT-sIMU, the framework was more likely to classify the athlete as elite than the ideal observer. A potential reason for this could be that some of the novice athletes were attending an elite youth sports academy, which boasts a high percentage of students continuing to compete at the collegiate and professional levels. Therefore, some of the novice athletes were on track to become elite athletes at the time of testing. On average, OMAT-OPT acted more closely to the ideal observer than OMAT-sIMU, based on our definition of a hit and correct rejection; this is represented by the smaller C value.

Although on average the models using the OMAT-sIMU data as the inputs, had higher classification rates than OMAT-OPT, a limitation of the OMAT-sIMU data is that it is more difficult to interpret differences between elites and novices compared to OMAT-OPT, making it harder to train individuals to improve their movement patterns. With only linear accelerations and angular velocities, and no video data, it is hard to discern exactly how the athlete is moving within space to obtain a score more representative of an elite or novice athlete. IMUs may offer an inexpensive measurement device to objectively screen movement abilities, where those individuals identified with weaknesses can then be tested more in depth with optical motion capture to inform targeted corrective exercise approaches.

A limitation of this study is the use of camera-based motion capture to calculate the linear accelerations and angular velocities for each segment and not raw data collected from IMUs. This technique has been used in previous research when the desired database does not contain IMU data (Young, 2010). Although linear accelerations and angular velocities would change when using IMUs, due to the inability to place IMUs at the CoG of segments, previous research has found strong agreement between IMU outputs and optical motion capture outputs (Mcginnis et al., 2014; Bolink et al., 2018). Even though the data were not raw data from an IMU, we purposefully took the Euclidean norm of the data to increase ecological validity and to remove the effect of different local coordinate system orientations of the linear acceleration and angular velocities within the global coordinate system. In addition, we differentiated positional data, which introduces noise to the data that would not be present when collecting data via IMU and were still able to get high classification rates. We are confident that these classification results are representative and may be lower than that of what would be achieved using sensors themselves, which we are in the process of testing. A second limitation of the study is the assumption athletes at the collegiate and professional level completed 10,000 h of deliberate practice. However, athletes competing at the professional and inter-collegiate levels would be in the higher echelon of athletes in their sport even if not completing 10,000 h. Nonetheless, this paper provides proof-of-concept that the OMAT is able to accurately classify athletes as novice or elite with consistent or improved accuracy when using data available from IMUs, relative to whole-body marker data. Future research should investigate the ability to classify athletes using OMAT using segment linear acceleration and angular velocity data collected using IMUs, fine-tuning algorithms to increase classification rates, and exploring other classifiers such as sport played, injury risk, and sex.

## Conclusion

In conclusion, the introduction of feature selection increased the classification rates compared to using the first 35 PC scores and BLR, LDA, and SVM produced the highest classification rates although there were minimal differences (<0.5%) between the three. Segment linear acceleration and angular velocity data readily available from an IMU could differentiate athletes’ movement performance based on skill level when using a novel machine learning approach (Ross et al., 2018) with a level of accuracy consistent with the use of whole-body motion capture data. These data suggest that IMUs, in conjunction with OMAT, may provide an inexpensive and timely way to objectively characterize and classify movement performance in the field, providing a feasible method for coaches and clinicians to objectively measure performance.

## Data Availability Statement

Sample code and data are available at: https://doi.org/10.5281/zenodo.3575075.

## Ethics Statement

The studies involving human participants were reviewed and approved by The Health Sciences and Science Research Ethics Board – University of Ottawa. Written informed consent to participate in this study was provided by the participant or if under the age of consent, by the participants’ legal guardian/next of kin.

## Author Contributions

GR, RG, NT, and SF conceived the study and interpreted the results. GR and BD collected the data and performed the pre-processing of the data. GR implemented the OMAT, analyzed the results, and prepared the manuscript. All authors revised the manuscript.

## Conflict of Interest

BD is the Director of Research at Motus. BD was involved in the data collection, pre-processing of the data, and editing of the manuscript and was not involved in the implementation of the OMAT or analysis of the results. In addition, the OMAT used in this paper is an objective, data driven approach with no human interaction and does not only apply to this data set. All other authors declare no potential conflict of interest.
